# 4-Chloro-7-methoxy­methyl-2-phenyl-7*H*-pyrrolo[2,3-*b*]pyridine

**DOI:** 10.1107/S1600536810008822

**Published:** 2010-03-13

**Authors:** Roland Selig, Dieter Schollmeyer, Wolfgang Albrecht, Stefan Laufer

**Affiliations:** a Eberhard-Karls-University Tübingen, Auf der Morgenstelle 8, 72076 Tübingen, Germany; b University Mainz, Duesbergweg 10-14, 55099 Mainz, Germany; c c-a-i-r biosciences GmbH, Paul-Ehrlich-Strasse 15, 72076 Tübingen, Germany

## Abstract

In the title compound, C_15_H_13_ClN_2_O, the phenyl group makes a dihedral angle of 7.91 (8)° with the pyrrole ring. The crystal structure forms a three-dimensional network stabilized by π–π inter­actions [centroid–centroid distances = 3.807 (1) Å] between the pyridine and phenyl rings and *via* inter­molecular C—H⋯O hydrogen bonds.

## Related literature

Chlorination of 2-phenyl-1*H*-pyrrolo[2,3-*b*]pyridine was performed by an analogous procedure, see: Layek *et al.* (2009 ▶).
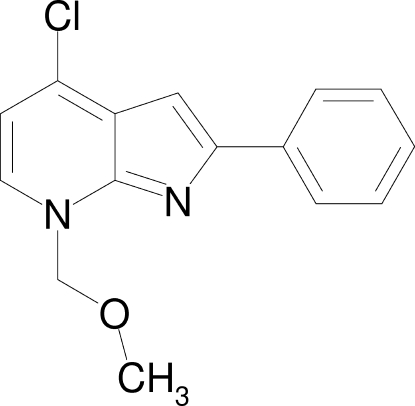


## Experimental

### 

#### Crystal data

C_15_H_13_ClN_2_O
*M* *_r_* = 272.72
Orthorhombic, 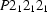

*a* = 8.4785 (8) Å
*b* = 9.6576 (10) Å
*c* = 15.8560 (16) Å
*V* = 1298.3 (2) Å^3^
*Z* = 4
Mo *K*α radiation
μ = 0.29 mm^−1^
*T* = 173 K
0.32 × 0.21 × 0.08 mm

#### Data collection

Bruker SMART APEXII diffractometer
5977 measured reflections
3084 independent reflections
2667 reflections with *I* > 2σ(*I*)
*R*_int_ = 0.028

#### Refinement

*R*[*F* ^2^ > 2σ(*F* ^2^)] = 0.037
*wR*(*F* ^2^) = 0.079
*S* = 1.03
3084 reflections
173 parameters
H-atom parameters constrained
Δρ_max_ = 0.21 e Å^−3^
Δρ_min_ = −0.22 e Å^−3^
Absolute structure: Flack (1983 ▶), 1299 Friedel pairs
Flack parameter: 0.02 (6)

### 

Data collection: *APEX2* (Bruker, 2006 ▶); cell refinement: *SAINT* (Bruker, 2006 ▶); data reduction: *SAINT*; program(s) used to solve structure: *SIR97* (Altomare *et al.*, 1999 ▶); program(s) used to refine structure: *SHELXL97* (Sheldrick, 2008 ▶); molecular graphics: *PLATON* (Spek, 2009 ▶); software used to prepare material for publication: *PLATON*.

## Supplementary Material

Crystal structure: contains datablocks I, global. DOI: 10.1107/S1600536810008822/bt5211sup1.cif

Structure factors: contains datablocks I. DOI: 10.1107/S1600536810008822/bt5211Isup2.hkl

Additional supplementary materials:  crystallographic information; 3D view; checkCIF report

## Figures and Tables

**Table 1:** Hydrogen-bond geometry (Å, °)

*D*—H⋯*A*	*D*—H	H⋯*A*	*D*⋯*A*	*D*—H⋯*A*
C5—H5⋯O15^i^	0.95	2.32	3.237 (2)	162

Symmetry code: (i) 
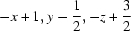
.

**Fig. 1.**
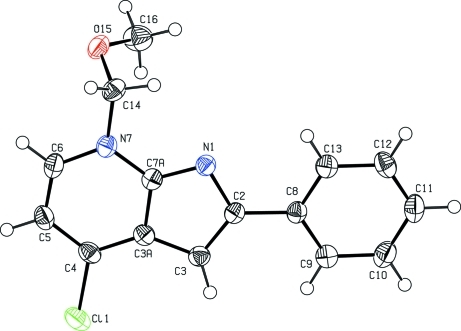
View of compound I. Displacement ellipsoids are drawn at the 50% probability level.

## supplementary crystallographic information

### Comment

N-protection of 7-azaindoles is a often used and necessary procedure for further NH sensitive reactions. Many protecting procedures with 4-chloro-1*H*-pyrrolo[2,3-b]pyridine are kown in literature. By N-protection of 4-chloro-2-phenyl-1*H*-pyrrolo[2,3-b]pyridine with methoxymethylchloride, two regioisomeres are formed, the expected 4-chloro-1-(methoxymethyl)-2-phenyl-1*H*-pyrrolo[2,3-b]pyridine and the title compound in a ratio of 1:1.6. The title compound and its regioisomer demonstrate the delocalization of the deprotonated anionic 4-chloro-1-(methoxymethyl)-2-phenyl-1*H*-pyrrolo[2,3-b]pyridine species. The phenyl moiety encloses a dihedral angle of 7.91 (8)° toward the azaindole system. The crystal structure is characterized by intermolecular hydrogen bond C5—H5···O15 (2.32 Å) and intramolecular hydrogen interactions C13—H13···N1 (2.54 Å), C14—H14B···N1 (2.48°). Stabilization of the three dimensional network is performed by π -π interactions between the pyridine and the phenyl rings with centroid distances of 3.807 (1) Å (symmetry operator 1.5-x, 1-y, -0.5∓z).

### Experimental

2,5 g (11 mmol) 4-chloro-1-(methoxymethyl)-2-phenyl-1*H*-pyrrolo[2,3-b]pyridine was dissolved in dry THF (15 ml). After addition of 0.61 g (15 mmol) NaH (60% in mineral oil) the reaction mixture was stirred for 15 minutes at room temperature. 7.3 ml (15 mmol) methoxymethylchloride (2.1M in toluene) was added and the mixture was stirred for further 15 minutes. The reaction mixture was quenched with concentrated aqueous ammonium chloride solution. After extraction with ethyl acetate, the crude product was purified by flash chromatography. Crystals suitable for X-ray analysis were obtained by slow crystallisation from methanol.

### Refinement

Hydrogen atoms attached to carbons were placed at calculated positions with C—H = 0.95 Å (aromatic) or 0.98–0.99 Å (*sp*^3^ C-atom). All H atoms were refined in the riding-model approximation with isotropic displacement parameters (set at 1.2–1.5 times of the *U*_eq_ of the parent atom).

### Crystal data

**Table:**

C_15_H_13_ClN_2_O	*F*(000) = 568
*M**_r_* = 272.72	*D*_x_ = 1.395 Mg m^−^^3^
Orthorhombic, *P*2_1_2_1_2_1_	Mo *K*α radiation, λ = 0.71073 Å
Hall symbol: P 2ac 2ab	Cell parameters from 1903 reflections
*a* = 8.4785 (8) Å	θ = 2.5–26.5°
*b* = 9.6576 (10) Å	µ = 0.29 mm^−^^1^
*c* = 15.8560 (16) Å	*T* = 173 K
*V* = 1298.3 (2) Å^3^	Block, yellow
*Z* = 4	0.32 × 0.21 × 0.08 mm

### Data collection

**Table:**

Bruker SMART APEXII diffractometer	2667 reflections with *I* > 2σ(*I*)
Radiation source: sealed tube	*R*_int_ = 0.028
graphite	θ_max_ = 27.9°, θ_min_ = 2.5°
CCD scan	*h* = −11→9
5977 measured reflections	*k* = −11→12
3084 independent reflections	*l* = −18→20

### Refinement

**Table:**

Refinement on *F*^2^	Secondary atom site location: difference Fourier map
Least-squares matrix: full	Hydrogen site location: inferred from neighbouring sites
*R*[*F*^2^ > 2σ(*F*^2^)] = 0.037	H-atom parameters constrained
*wR*(*F*^2^) = 0.079	*w* = 1/[σ^2^(*F*_o_^2^) + (0.0335*P*)^2^ + 0.0915*P*] where *P* = (*F*_o_^2^ + 2*F*_c_^2^)/3
*S* = 1.03	(Δ/σ)_max_ < 0.001
3084 reflections	Δρ_max_ = 0.21 e Å^−^^3^
173 parameters	Δρ_min_ = −0.22 e Å^−^^3^
0 restraints	Absolute structure: Flack (1983), 1294 Friedel pairs
Primary atom site location: structure-invariant direct methods	Flack parameter: 0.02 (6)

### Special details

**Table:**

Geometry. All esds (except the esd in the dihedral angle between two l.s. planes) are estimated using the full covariance matrix. The cell esds are taken into account individually in the estimation of esds in distances, angles and torsion angles; correlations between esds in cell parameters are only used when they are defined by crystal symmetry. An approximate (isotropic) treatment of cell esds is used for estimating esds involving l.s. planes.
Refinement. Refinement of *F*^2^ against ALL reflections. The weighted *R*-factor wR and goodness of fit *S* are based on *F*^2^, conventional *R*-factors *R* are based on *F*, with *F* set to zero for negative *F*^2^. The threshold expression of *F*^2^ > σ(*F*^2^) is used only for calculating *R*-factors(gt) etc. and is not relevant to the choice of reflections for refinement. *R*-factors based on *F*^2^ are statistically about twice as large as those based on *F*, and *R*- factors based on ALL data will be even larger.

### Fractional atomic coordinates and isotropic or equivalent isotropic displacement parameters (Å^2^)

**Table:**

	*x*	*y*	*z*	*U*_iso_*/*U*_eq_	
Cl1	0.29645 (6)	0.30454 (5)	0.60634 (3)	0.03778 (14)	
N1	0.64025 (18)	0.65066 (16)	0.45780 (9)	0.0247 (3)	
C2	0.5784 (2)	0.55036 (19)	0.40408 (11)	0.0237 (4)	
C3	0.4866 (2)	0.4524 (2)	0.44513 (11)	0.0258 (4)	
H3	0.4352	0.3746	0.4208	0.031*	
C3A	0.4856 (2)	0.4927 (2)	0.53056 (11)	0.0245 (4)	
C4	0.4173 (2)	0.44943 (18)	0.60500 (12)	0.0270 (4)	
C5	0.4472 (2)	0.5205 (2)	0.68003 (11)	0.0300 (4)	
H5	0.3997	0.4909	0.7312	0.036*	
C6	0.5454 (2)	0.6330 (2)	0.67921 (11)	0.0299 (4)	
H6	0.5669	0.6793	0.7308	0.036*	
N7	0.61298 (17)	0.68081 (16)	0.60720 (9)	0.0263 (3)	
C7A	0.5837 (2)	0.61451 (19)	0.53258 (11)	0.0242 (4)	
C8	0.6076 (2)	0.56108 (19)	0.31251 (10)	0.0246 (4)	
C9	0.5316 (2)	0.4728 (2)	0.25586 (13)	0.0328 (5)	
H9	0.4647	0.4017	0.2767	0.039*	
C10	0.5525 (3)	0.4874 (2)	0.16978 (12)	0.0367 (5)	
H10	0.4995	0.4268	0.1321	0.044*	
C11	0.6500 (3)	0.5900 (2)	0.13843 (12)	0.0349 (5)	
H11	0.6630	0.6008	0.0793	0.042*	
C12	0.7285 (2)	0.6765 (2)	0.19367 (12)	0.0329 (5)	
H12	0.7980	0.7453	0.1725	0.040*	
C13	0.7061 (2)	0.6632 (2)	0.28038 (11)	0.0289 (4)	
H13	0.7587	0.7245	0.3178	0.035*	
C14	0.7186 (2)	0.8020 (2)	0.61091 (11)	0.0305 (4)	
H14A	0.8005	0.7861	0.6544	0.037*	
H14B	0.7723	0.8131	0.5559	0.037*	
O15	0.63663 (17)	0.92266 (14)	0.62990 (7)	0.0332 (3)	
C16	0.5440 (3)	0.9729 (2)	0.56147 (14)	0.0435 (6)	
H16A	0.4921	1.0594	0.5781	0.065*	
H16B	0.4640	0.9039	0.5466	0.065*	
H16C	0.6123	0.9901	0.5127	0.065*	

### Atomic displacement parameters (Å^2^)

**Table:**

	*U*^11^	*U*^22^	*U*^33^	*U*^12^	*U*^13^	*U*^23^
Cl1	0.0405 (3)	0.0307 (2)	0.0422 (3)	−0.0039 (2)	0.0068 (2)	0.0094 (2)
N1	0.0236 (8)	0.0267 (8)	0.0239 (8)	−0.0023 (7)	0.0015 (6)	−0.0018 (6)
C2	0.0219 (9)	0.0252 (9)	0.0241 (8)	0.0029 (8)	−0.0003 (7)	−0.0015 (7)
C3	0.0272 (10)	0.0230 (9)	0.0272 (9)	0.0013 (8)	−0.0015 (7)	−0.0004 (7)
C3A	0.0233 (9)	0.0239 (9)	0.0262 (9)	0.0038 (8)	−0.0022 (7)	0.0026 (7)
C4	0.0257 (9)	0.0239 (9)	0.0313 (9)	0.0040 (8)	0.0024 (8)	0.0059 (8)
C5	0.0341 (11)	0.0309 (11)	0.0251 (10)	0.0071 (9)	0.0047 (8)	0.0063 (8)
C6	0.0337 (11)	0.0344 (11)	0.0215 (9)	0.0066 (9)	−0.0002 (8)	−0.0019 (7)
N7	0.0258 (8)	0.0294 (8)	0.0237 (7)	0.0014 (7)	−0.0018 (6)	−0.0014 (7)
C7A	0.0222 (9)	0.0261 (9)	0.0242 (9)	0.0026 (8)	−0.0022 (7)	0.0000 (7)
C8	0.0242 (10)	0.0260 (10)	0.0238 (8)	0.0052 (8)	0.0013 (7)	−0.0008 (7)
C9	0.0377 (12)	0.0317 (11)	0.0290 (10)	−0.0023 (9)	0.0015 (8)	0.0001 (8)
C10	0.0430 (13)	0.0395 (12)	0.0276 (10)	0.0049 (11)	−0.0036 (8)	−0.0056 (8)
C11	0.0397 (12)	0.0418 (12)	0.0233 (9)	0.0141 (10)	0.0025 (8)	0.0027 (8)
C12	0.0301 (11)	0.0364 (11)	0.0323 (10)	0.0053 (10)	0.0070 (8)	0.0084 (8)
C13	0.0259 (10)	0.0311 (10)	0.0297 (9)	0.0020 (9)	0.0013 (8)	−0.0005 (7)
C14	0.0276 (10)	0.0328 (9)	0.0311 (9)	−0.0026 (10)	−0.0024 (8)	−0.0067 (9)
O15	0.0412 (8)	0.0321 (7)	0.0263 (7)	0.0032 (7)	0.0014 (5)	−0.0073 (5)
C16	0.0458 (14)	0.0366 (13)	0.0480 (13)	0.0034 (12)	−0.0096 (10)	0.0018 (10)

### Geometric parameters (Å, °)

**Table:**

Cl1—C4	1.7345 (19)	C8—C9	1.396 (3)
N1—C7A	1.326 (2)	C9—C10	1.383 (3)
N1—C2	1.393 (2)	C9—H9	0.9500
C2—C3	1.387 (3)	C10—C11	1.383 (3)
C2—C8	1.476 (2)	C10—H10	0.9500
C3—C3A	1.409 (2)	C11—C12	1.381 (3)
C3—H3	0.9500	C11—H11	0.9500
C3A—C4	1.379 (3)	C12—C13	1.394 (3)
C3A—C7A	1.441 (3)	C12—H12	0.9500
C4—C5	1.396 (3)	C13—H13	0.9500
C5—C6	1.369 (3)	C14—O15	1.390 (2)
C5—H5	0.9500	C14—H14A	0.9900
C6—N7	1.358 (2)	C14—H14B	0.9900
C6—H6	0.9500	O15—C16	1.425 (2)
N7—C7A	1.368 (2)	C16—H16A	0.9800
N7—C14	1.475 (2)	C16—H16B	0.9800
C8—C13	1.389 (3)	C16—H16C	0.9800
			
C7A—N1—C2	103.15 (15)	C10—C9—C8	120.91 (19)
C3—C2—N1	113.49 (16)	C10—C9—H9	119.5
C3—C2—C8	127.11 (16)	C8—C9—H9	119.5
N1—C2—C8	119.31 (16)	C11—C10—C9	120.27 (19)
C2—C3—C3A	105.44 (16)	C11—C10—H10	119.9
C2—C3—H3	127.3	C9—C10—H10	119.9
C3A—C3—H3	127.3	C12—C11—C10	119.57 (18)
C4—C3A—C3	137.84 (18)	C12—C11—H11	120.2
C4—C3A—C7A	118.06 (16)	C10—C11—H11	120.2
C3—C3A—C7A	104.08 (15)	C11—C12—C13	120.28 (19)
C3A—C4—C5	120.28 (17)	C11—C12—H12	119.9
C3A—C4—Cl1	120.18 (14)	C13—C12—H12	119.9
C5—C4—Cl1	119.54 (14)	C8—C13—C12	120.59 (17)
C6—C5—C4	119.48 (17)	C8—C13—H13	119.7
C6—C5—H5	120.3	C12—C13—H13	119.7
C4—C5—H5	120.3	O15—C14—N7	111.71 (14)
N7—C6—C5	122.29 (17)	O15—C14—H14A	109.3
N7—C6—H6	118.9	N7—C14—H14A	109.3
C5—C6—H6	118.9	O15—C14—H14B	109.3
C6—N7—C7A	119.43 (16)	N7—C14—H14B	109.3
C6—N7—C14	119.50 (15)	H14A—C14—H14B	107.9
C7A—N7—C14	121.07 (15)	C14—O15—C16	113.35 (14)
N1—C7A—N7	125.76 (17)	O15—C16—H16A	109.5
N1—C7A—C3A	113.83 (15)	O15—C16—H16B	109.5
N7—C7A—C3A	120.40 (15)	H16A—C16—H16B	109.5
C13—C8—C9	118.36 (17)	O15—C16—H16C	109.5
C13—C8—C2	120.75 (16)	H16A—C16—H16C	109.5
C9—C8—C2	120.84 (17)	H16B—C16—H16C	109.5
			
C7A—N1—C2—C3	−0.8 (2)	C14—N7—C7A—C3A	177.31 (16)
C7A—N1—C2—C8	176.10 (15)	C4—C3A—C7A—N1	−178.06 (17)
N1—C2—C3—C3A	1.3 (2)	C3—C3A—C7A—N1	0.7 (2)
C8—C2—C3—C3A	−175.36 (16)	C4—C3A—C7A—N7	2.8 (3)
C2—C3—C3A—C4	177.3 (2)	C3—C3A—C7A—N7	−178.39 (16)
C2—C3—C3A—C7A	−1.13 (19)	C3—C2—C8—C13	−178.33 (18)
C3—C3A—C4—C5	180.0 (2)	N1—C2—C8—C13	5.2 (3)
C7A—C3A—C4—C5	−1.8 (3)	C3—C2—C8—C9	4.5 (3)
C3—C3A—C4—Cl1	1.1 (3)	N1—C2—C8—C9	−171.98 (17)
C7A—C3A—C4—Cl1	179.34 (13)	C13—C8—C9—C10	−0.7 (3)
C3A—C4—C5—C6	−0.3 (3)	C2—C8—C9—C10	176.57 (18)
Cl1—C4—C5—C6	178.58 (14)	C8—C9—C10—C11	0.3 (3)
C4—C5—C6—N7	1.5 (3)	C9—C10—C11—C12	1.0 (3)
C5—C6—N7—C7A	−0.4 (3)	C10—C11—C12—C13	−1.8 (3)
C5—C6—N7—C14	−179.51 (17)	C9—C8—C13—C12	−0.2 (3)
C2—N1—C7A—N7	179.07 (17)	C2—C8—C13—C12	−177.44 (17)
C2—N1—C7A—C3A	0.0 (2)	C11—C12—C13—C8	1.4 (3)
C6—N7—C7A—N1	179.26 (18)	C6—N7—C14—O15	−68.9 (2)
C14—N7—C7A—N1	−1.7 (3)	C7A—N7—C14—O15	111.99 (17)
C6—N7—C7A—C3A	−1.8 (3)	N7—C14—O15—C16	−73.6 (2)

### Hydrogen-bond geometry (Å, °)

**Table:**

*D*—H···*A*	*D*—H	H···*A*	*D*···*A*	*D*—H···*A*
C5—H5···O15^i^	0.95	2.32	3.237 (2)	162

Symmetry codes: (i) −*x*+1, *y*−1/2, −*z*+3/2.
